# *In vivo* microscopic voxel-based morphometry with a brain template to characterize strain-specific structures in the mouse brain

**DOI:** 10.1038/s41598-017-00148-1

**Published:** 2017-03-07

**Authors:** Keigo Hikishima, Yuji Komaki, Fumiko Seki, Yasuyuki Ohnishi, Hirotaka J. Okano, Hideyuki Okano

**Affiliations:** 1 Department of Physiology, Keio University School of Medicine, Tokyo, Japan; 2 Central Institute for Experimental Animals, Kawasaki, Japan; 3 Okinawa Institute of Science and Technology Graduate University, Okinawa, Japan; 4 Laboratory for Marmoset Neural Architecture, Brain Science Institute RIKEN, Wako, Japan; 5 Division of Regenerative Medicine, Jikei University School of Medicine, Tokyo, Japan

## Abstract

Hundreds of inbred mouse strains are established for use in a broad spectrum of basic research fields, including genetics, neuroscience, immunology, and cancer. Inbred mice exhibit identical intra-strain genetics and divergent inter-strain phenotypes. The cognitive and behavioral divergences must be controlled by the variances of structure and function of their brains; however, the underlying morphological features of strain-to-strain difference remain obscure. Here, *in vivo* microscopic magnetic resonance imaging was optimized to image the mouse brains by using an isotropic resolution of 80 μm. Next, *in vivo* templates were created from the data from four major inbred mouse strains (C57Bl/6, BALB/cBy, C3H/He, and DBA/2). A strain-mixed brain template was also created, and the template was then employed to establish automatic voxel-based morphometry (VBM) for the mouse brain. The VBM assessment revealed strain-specific brain morphologies concerning the gray matter volume of the four strains, with a smaller volume in the primary visual cortex for the C3H/He strain, and a smaller volume in the primary auditory cortex and field CA1 of the hippocampus for the DBA/2 strain. These findings would contribute to the basis of for understanding morphological phenotype of the inbred mouse strain and may indicate a relationship between brain morphology and strain-specific cognition and behavior.

## Introduction

Numerous mouse strains are currently available for use in a wide range of research fields, including genetics, neuroscience, immunology, and cancer. Hundreds of strains have been established for inbred mice alone. These inbred animals exhibit identical intra-strain genetics along with notable inter-strain phenotypic differences (e.g. hair color, behavior, and carcinogenicity). The genetic uniformity of inbred mice of the same strain permits the study of inter-strain phenotypes diversity, focusing on cognitive and behavioral differences and genetic background among assorted strains over long periods of time^1^. The differences between strains should be based on distinct structures and functions of their brains; however, it remains unclear whether specific structural modifications are responsible for the observed phenotypic discrepancies.

Magnetic resonance imaging (MRI) is an imaging modality widely used in clinical practice. Voxel-based morphometry (VBM) registers groups of individual MRI-obtained brain morphologies into a standard brain template, and then analyzes differences in brain morphology between groups by using voxel-wise statistics^2^. With the aid of VBM, it is possible to characterize patterns of brain atrophy in various neurological and psychiatric diseases, including Alzheimer’s disease, mild cognitive impairment, autism, and schizophrenia^3, 4^.

Since the mouse brain (0.5 cm^3^) is 2800 times smaller than the human brain (1400 cm^3^), a microscopic voxel size smaller than 100 μm matches the voxel size used for VBM analysis in the human brain. To accomplish such microscopic resolution in mice, voxel-based analysis (VBA), including VBM of the mouse brain, has been evaluated *ex vivo* ^5–11^.

Recently, MRI hardware has become more sophisticated. In particular, cryogenic coils are now available for mice, and *in vivo* measurements of the mouse brain can be realized at extremely high spatial resolution^12^. Indeed, *in vivo* VBM of the mouse brain has been used to detect increased hippocampal volume during physical exercise^13^.

In this study, we obtained *in vivo* T1-weighted MR images with an isotropic spatial resolution of 80 μm from four major inbred mouse strains: C57Bl/6, BALB/cBy, C3H/He, and DBA/2. We then created brain templates from these strains to automatically analyze the morphology of the mouse brain. Additionally, we created a strain-mixed template and used the brain template to conduct a VBM analysis of all four strains, with the goal of discriminating differences in brain morphology among them.

## Materials and Methods

### Animals

In total, 60 male mice (C57Bl/6, n = 30; BALB/cBy, n = 10; C3H/He, n = 10; and DBA/2, n = 10) were provided by CLEA Japan, Inc. (Tokyo, Japan) and used in the current study. All procedures were performed in accordance with the Laboratory Animal Welfare Act and the Guide for the Care and Use of Laboratory Animals (National Institutes of Health, Bethesda, MD, USA). The current protocol was reviewed by the Institutional Animal Care and Use Committee and approved by the Central Institute for Experimental Animals (CIEA) of Japan (CIEA Approval No. 10037A; CIEA, Kawasaki, Japan). All mice were housed under conditions of a 12 h light/dark cycle and provided with free access to food and water.

### MRI

*In vivo* MRI was performed by using a seven-tesla (7-T) Biospec 70/16 MRI system equipped with actively shielded gradients at a maximum strength of 700 mT/m (Bruker Biospec GmbH, Ettlingen, Germany) and a cryogenic quadrature RF surface probe (CryoProbe; Bruker BioSpin AG, Fällanden, Switzerland). Mice (17-weeks-old) were initially anesthetized with 4.0% isoflurane in air. Experiments were carried out at a maintenance anesthesia level of 1.5%. High-resolution T1-weighted imaging data were acquired by using an optimized MPRAGE (magnetization-prepared rapid gradient-echo) sequence^14^. The imaging parameters were as follows: repetition time/echo time = 11.8/3.7 ms; inversion time = 1300 ms; time to delay = 3700 ms; field of view = 15.4 × 12.8 × 7.4 mm on a 192 × 160 × 92 matrix; number of segments = 3; and number of averages = 4. The total scan time was 2 h. Next, the obtained images were de-noised with a 3D non-local means (NLM) filter implemented within Amira version 5.4 (FEI Visualization Sciences Group, Burlington, MA) for VBM analysis, which helps the segmentation process by removing noise while preserving edges^15^. Finally, the voxel size was multiplied by 10 for use directly in SPM software (Wellcome Trust Centre for Neuroimaging, UCL Institute of Neurology, London, UK; www.fil.ion.ucl.ac.uk/spm).

### Template creation

The brain template for the four inbred strains was created as follows^16^:

- Skull-stripping.
  The Brain Extraction Tool (BET) is commonly used for automatic brain extraction in human MRI data^17^. However, use of this tool with the current mouse MRI data resulted in deficits in the frontal and occipital regions due to anatomical differences between mice and humans. Therefore, skull-stripping was manually performed to obtain accurate brain extractions by using the “Segmentation Editor” tool in the Amira software package, version 5.2 (Visage Imaging, Inc., San Diego, CA, USA).
- Registration to a standard stereotaxic mouse brain.
  The post-mortem brain template of the SPM Mouse (http://www.spmmouse.org/) was utilized as the stereotaxic reference image. An angle of posterior rotation was determined to re-slice the *in vivo* MRI data. To do this, affine registration of the *in vivo* MRI to the post-mortem template was performed by applying the “Affine Registration” tool with a rigid body model in Amira. Next, the *in vivo* images were re-sliced by using the “Apply Transform” function to obtain accurate stereotaxic coordinates of the brain.
- Segmentation and template creation.

A two-stage process was used to segment the head images into gray matter (GM), white matter (WM), cerebrospinal fluid (CSF), tissue outside the brain, and air, as follows. First, the “Segmentation” tool in SPM8 was used for all skull-stripped mouse brain data. The data were divided into GM, WM, and CSF images using a mixture of Gaussians and tissue probability maps (TPMs) of the SPM Mouse. An *in vivo* brain template was then created by using the DARTEL toolbox (Ashburner, 2007) in SPM8, which improves registration with an inverse consistent, diffeomorphic transformation. Next, the *in vivo* brain template was employed to average each set of data for tissue outside the brain and air as probability maps for segmentation, respectively. DARTEL in SPM8 was also used to finally complete an *in vivo* brain template, including tissue outside the brain and air.

The bounding box (a three-dimensional (3D) space) encompassed the following x, y, and z dimensions and the origin in mm: x = −6.8, 6.8; y = −9.8, 7.4; z = −8.7, 2.2; and origin = 0, 0, 0. The horizontal slice (z = 0) passed through the height of the Bregma, while the coronal slice (y = 0) lay perpendicular to the horizontal plane and passed through the Bregma. The sagittal slice (x = 0) was located at the median sagittal plane. The stereotaxic, population-averaged, tissue-segmented brain templates created from the C57Bl/6, BALB/cBy, C3H/H3, and DBA/2 mice have been made freely available (http://www.nitrc.org/projects/tpm_mouse).

### VBM analysis

A strain-mixed brain template was created from four inbred mice (C57Bl/6, BALB/cBy, C3H/He, and DBA/2), and unified segmentation in SPM8 was applied along with the strain-mixed template to T1WI for all four inbred strains (n = 10 × 4). Modulated normalization images of the GM were obtained with DARTEL, multiplied by the Jacobian determinants derived from the spatial normalization^18^, and used to evaluate the local tissue volume of the GM. The images were spatially smoothed by convolving with an isotropic Gaussian kernel (full width at half maximum) of five times the voxel size to minimize the risk of false positives in statistical analysis.

The differences in GM volume (C57Bl/6 *vs.* BALB/cBy *vs.* C3H/He *vs.* DBA/2) were statistically assessed using a factorial model for a 1 × 4 analysis of variance (ANOVA), with the total brain volume as the covariate of no interest in SPM8. The significance level was set at p < 0.05 (family-wise error-corrected based on voxel-wise smoothness estimated from random field theory), and the extent threshold for cluster size was set at k > 119 (based on the expected number of voxels per cluster (k = 118.6)).

## Results

### Template creation

**Figure 1:**
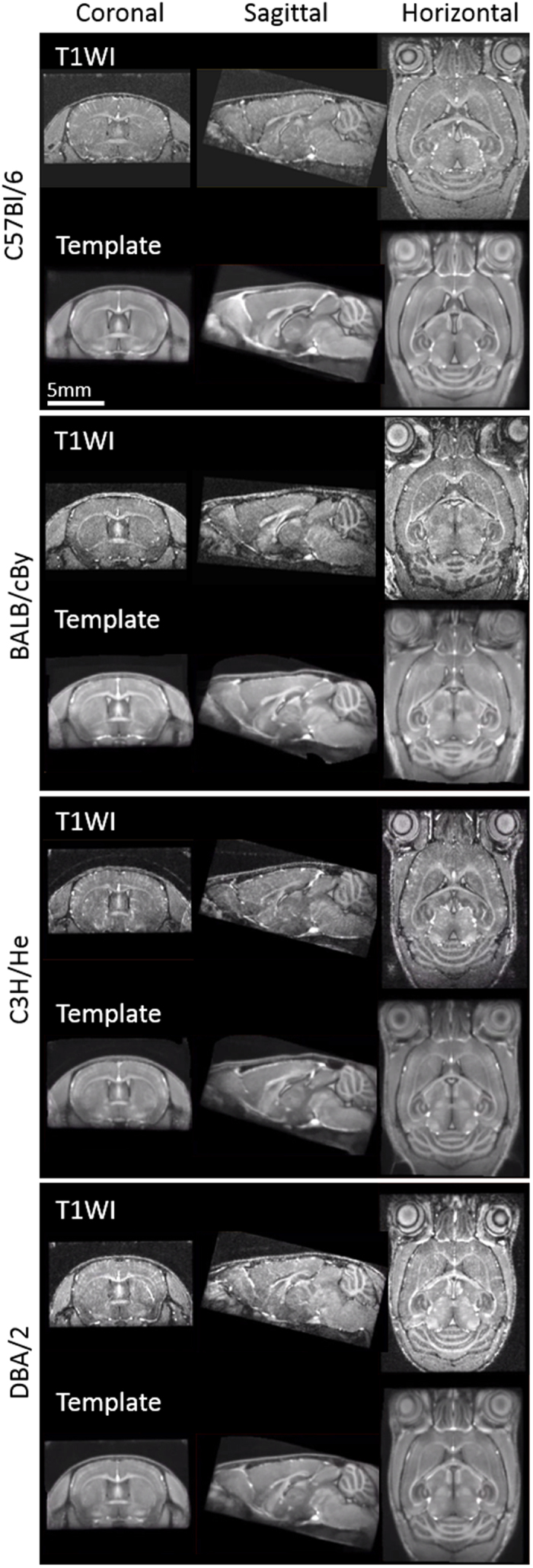
*In vivo* microscopic T1WI and templates of the mouse head in four major inbred strains. Coronal, sagittal, and horizontal sections of stereotaxic single and template T1WI of C57Bl/6, BALB/cBy, C3H/He, and DBA/2 mouse heads.

**Figure 2:**
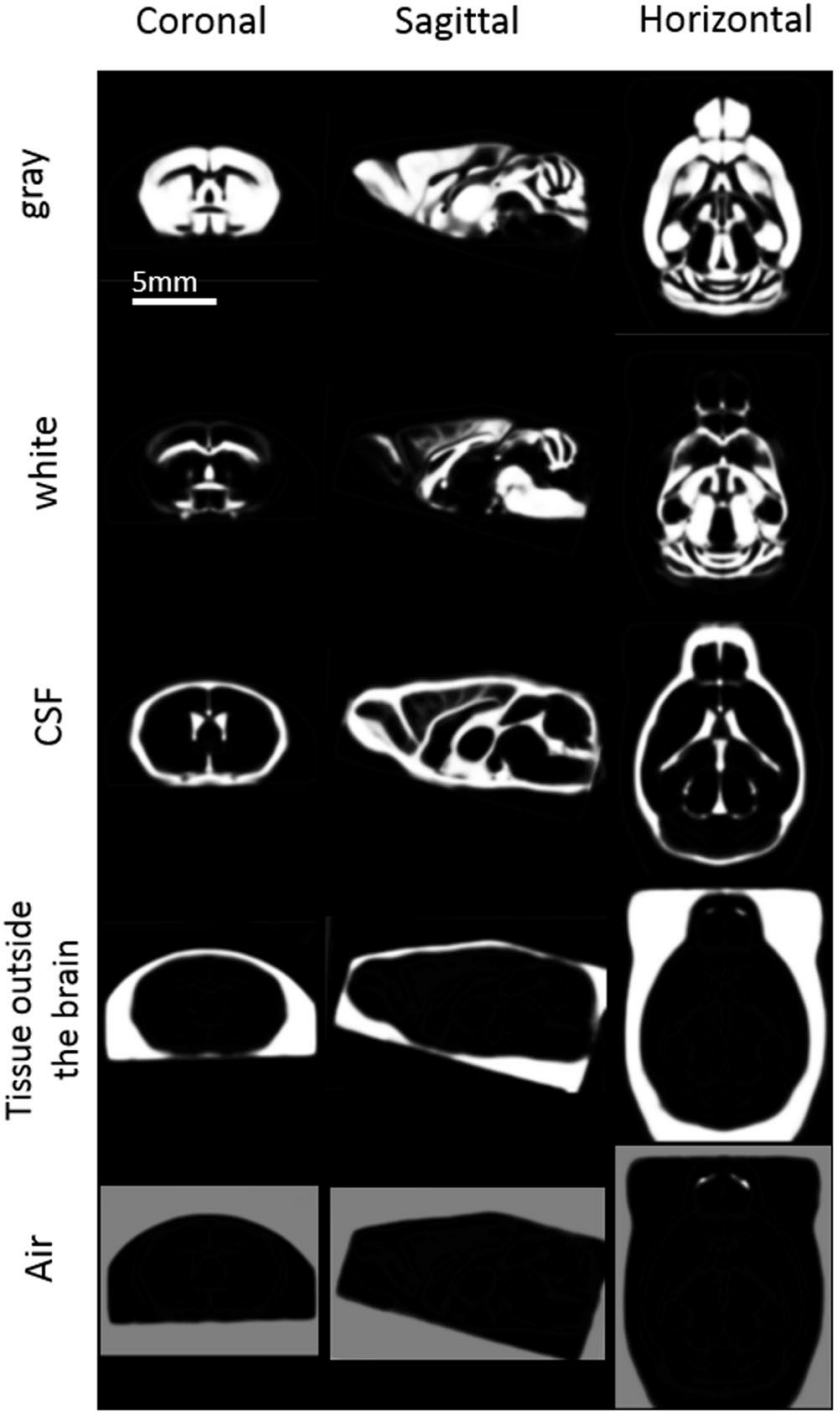
Brain tissue probability maps of C57Bl/6 mice. Coronal, sagittal, and horizontal sections of tissue probability maps of gray matter (GM), white matter (WM), cerebrospinal fluid (CSF), tissue outside the brain, and air.

Based on the *in vivo* microscopic T1WIs for the C57Bl/6, BALB/cBy, C3H/He, and DBA/2 mouse brains, we created a population-averaged standard brain template for each strain (Fig. 1), including TPMs (C57Bl/6 in Fig. 2). All of these templates are freely available (http://www.nitrc.org/projects/tpm_mouse). The differences between coordinates in selected landmarks of the template and a photomicrographic atlas by Franklin & Paxinos^19^ are shown in Table 1; there was a relatively small average difference of 0.09 mm in the x direction, 0.02 mm in the y direction and 0.08 mm in the z direction.

**Table 1:** Stereotaxic coordinates of major landmarks in template and a photomicrographic atlas by Franklin & Paxinos (2008).

Landmarks	MRI template			Photomicrographic by Franklin & Paxinos				Differences between template and atlas		
	X	Y	Z	X	Y	Z		X	Y	Z
CC in y = 0.00 mm	0.00	0.00	−2.02	0.0	0.00	−2.04		—	—	0.02
CC in y = −2.00 mm	0.00	−2.00	−1.38	0.0	−2.00	−1.55		—	—	0.17
AC	0.00	0.04	−4.10	0.0	0.02	−4.18		—	0.02	0.08
L sup. CPu in y = 0.86 mm	1.52	0.86	−2.18	1.43	0.86	−2.24		0.11	—	0.06
R sup. CPu in y = 0.86 mm	−1.44	0.86	−2.18	−1.38	0.86	−2.27		0.06	—	0.09
L lat. CPu in y = 0.86 mm	2.56	0.86	−3.46	2.65	0.86	−3.40		0.09	—	0.06
R lat. CPu in y = 0.86 mm	−2.56	0.86	−3.46	−2.64	0.86	−3.56		0.08	—	0.10
							average	0.09	0.02	0.08

The landmarks were the carpus callosum (CC) in two points (Y = 0, −2); the anterior commissure (AC); sup. CPu, superior extent of the caudate putamen; lat. Caudate, lateral extent of the caudate putamen in anterior one point (Y = 0.86).

All measurements are in mm and in stereotaxic coordinates from origin (X, Y, Z = 0, 0, 0) at the bregma.

### Differences in brain morphology among strains

**Figure 3:**
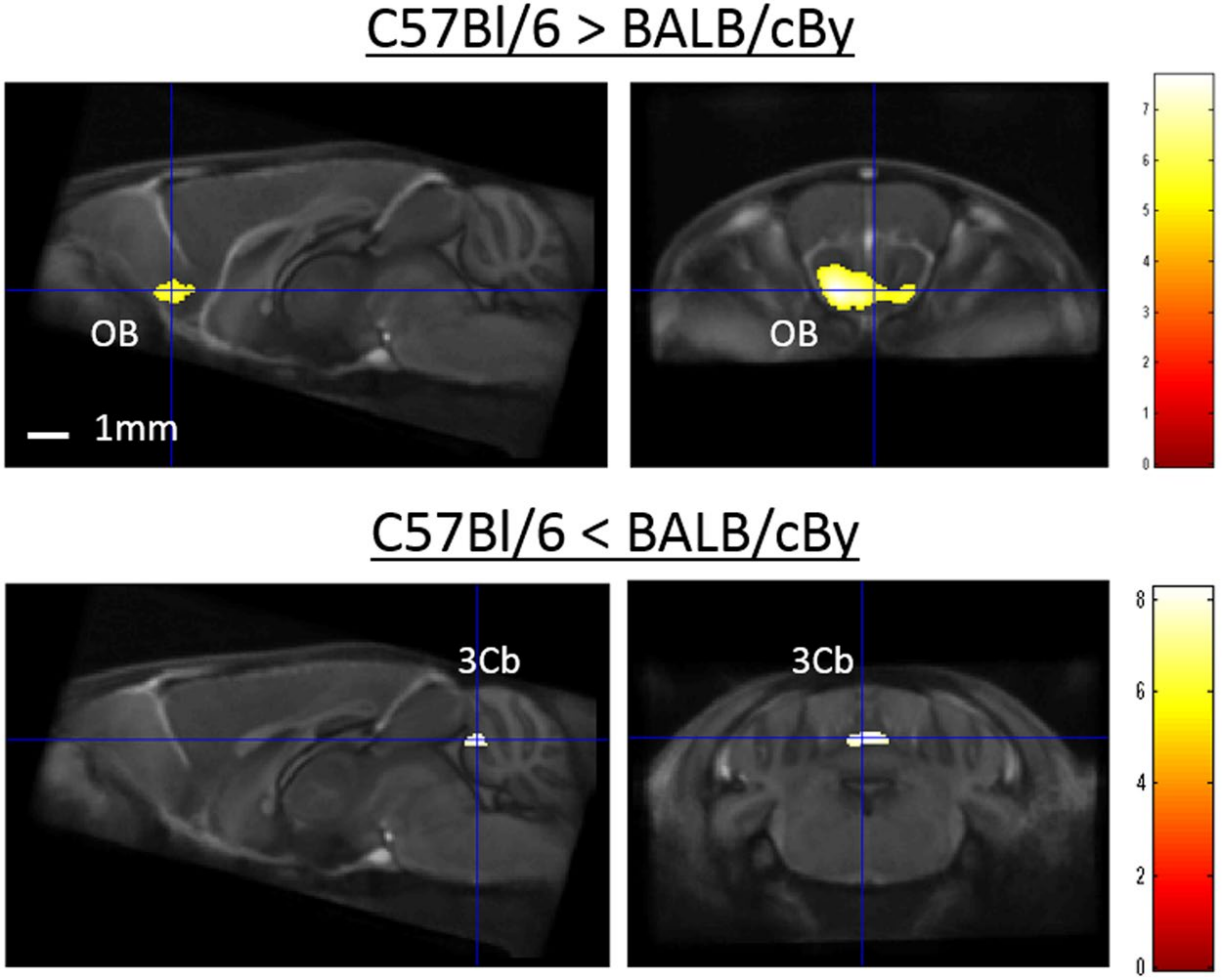
Representative depiction of volume differences in gray matter between C57Bl/6 and BALB/cBy mouse strains. Voxel-based morphometry revealed that the olfactory bulb (OB) of C57Bl/6 mice was significantly greater than that of BALB/cBy mice, and that lobule 3 of the cerebellar vermis (3Cb) of BALB/cBy mice was significantly greater than that of C57Bl/6 mice. The color scale indicates the t-score (p < 0.05, family-wise error-corrected), for which significant areas were superimposed on a population-average of all strains (standard template brain).

To investigate strain-specific brain morphology in the four inbred strains (C57Bl/6, BALB/cBy, C3H/He, and DBA/2), we conducted a VBM analysis of GM volume, which allowed pairwise comparison of two strains. For example, a comparison of the C57Bl/6 and BALB/cBy strains demonstrated significantly greater GM volume in the olfactory bulb (OB) of C57Bl/6 mice (left: x = 0.96, y = 2.76, z = −3.94, Z-score = 4.7; and right: x = −0.88, y = 2.68, z = −3.70, Z-score = 5.8) and significantly greater GM volume in lobule 3 of the cerebellar vermis (3Cb) of BALB/cBy mice (x = 0.08, y = −6.04, z = −2.18, Z-score = 4.8) (Fig. 3).

**Figure 4:**
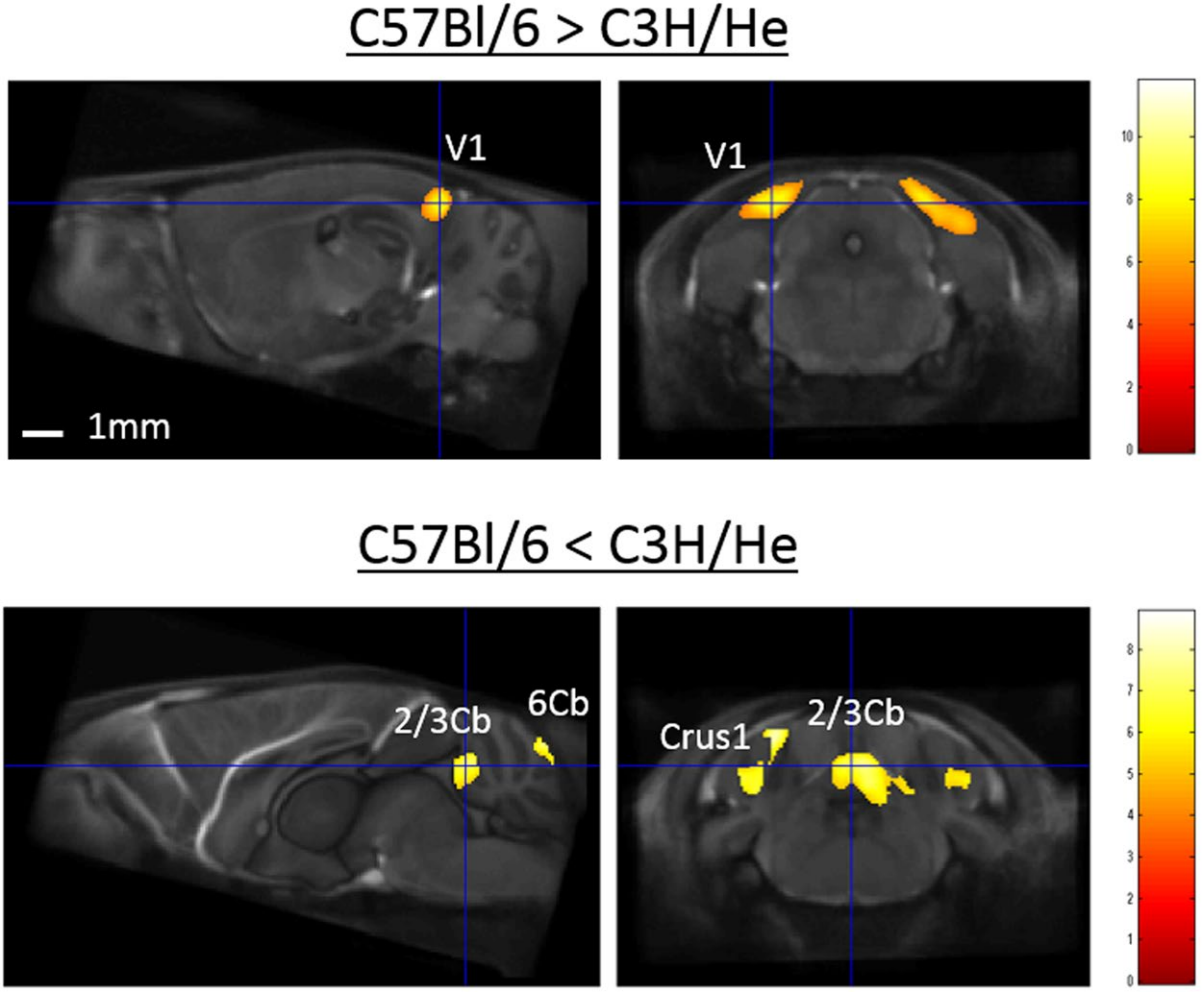
Representative depiction of volume differences in gray matter between C57Bl/6 and C3H/He mouse strains. Voxel-based morphometry revealed that the primary visual cortex (V1) of C57Bl/6 mice was significantly greater than that of C3H/He mice, and that lobules 2 and 3 of the cerebellar vermis (2/3Cb) and crus 1 of the ansiform lobule (Crus1) of C3H/He mice were significantly greater than those of C57Bl/6 mice. The color scale indicates the t-score (p < 0.05, family-wise error-corrected), for which significant areas were superimposed on a population-average of all strains (standard template brain).

A comparison of the C57Bl/6 and C3H/He strains demonstrated significantly greater GM volume in the bilateral primary visual cortex (V1) of C57Bl/6 mice (left: x = 2.00, y = −5.16, z = −1.14, Z-score = 5.8; and right: x = −2.40, y = −5.16, z = −1.30, Z-score = 6.1) and significantly greater GM volume in the many cerebellar cortices (lobule 6 of the cerebellar vermis (6Cb), lobules 2 and 3 of the cerebellar vermis (2/3Cb), simple lobules (Sim), and crus 2 of the ansiform lobule (Crus2)) of C3H/He mice (Fig. 4).

**Figure 5:**
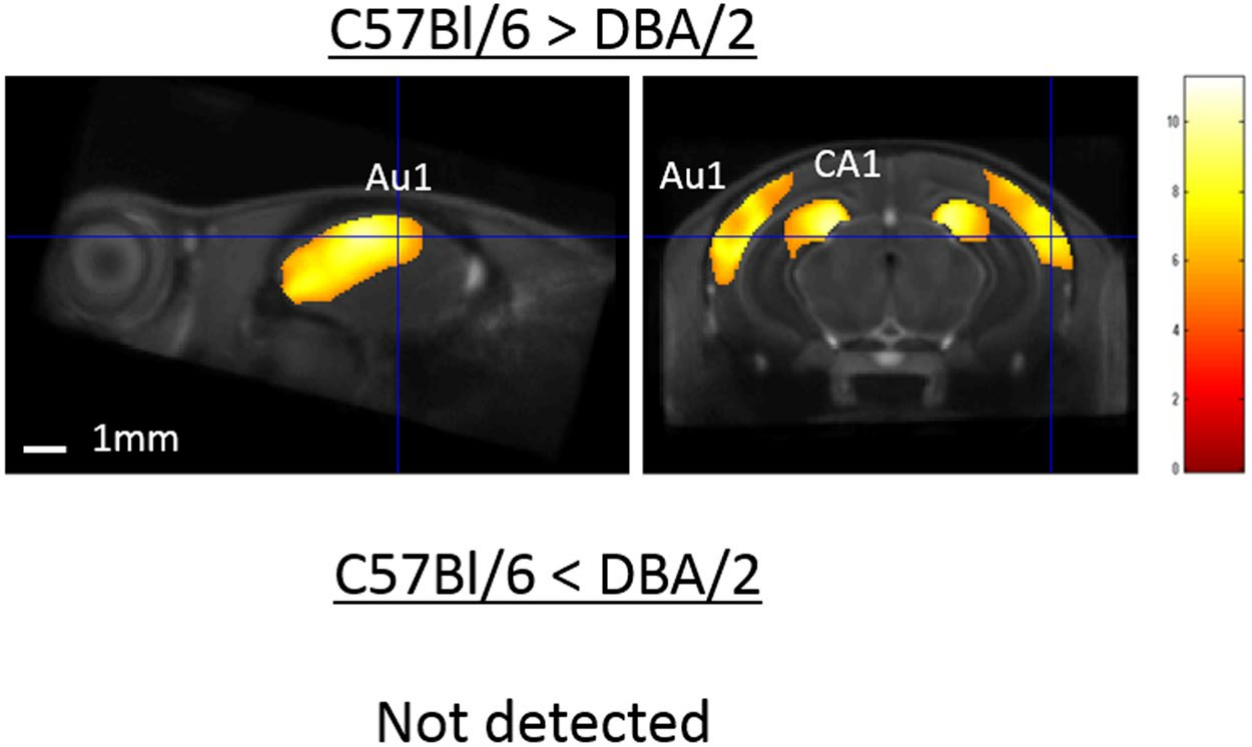
Representative depiction of volume differences in gray matter between C57Bl/6 and DBA/2 mouse strains. Voxel-based morphometry revealed that the primary auditory cortex (Au1) and field CA1 of the hippocampus of C57Bl/6 mice were significantly greater than those of DBA/2 mice. There were no regions in DBA/2 mice that were greater than those of C57Bl/6 mice. The color scale indicates the t-score (p < 0.05, family-wise error-corrected), for which significant areas were superimposed on a population-average of all strains (standard template brain).

A comparison of the C57Bl/6 and DBA/2 strains demonstrated significantly greater GM volume in the bilateral primary auditory cortex (Au1) (left: x = 4.56, y = −2.60, z = −2.10, Z-score = 6.8; and right: x = −4.08, y = −2.92, z = −2.50, Z-score = 6.7) and the bilateral field CA1 of the hippocampus (left: x = 1.52, y = −3.16, z = −1.62, Z-score = 6.9; and right: x = −1.84, y = −3.56, z = −2.02, Z-score = 6.9) of C57Bl/6 mice (Fig. 5). There was no significant difference between the GM region of DBA/2 mice and that of C57Bl/6 mice.

Comparison of all four strains (see Table S1) revealed that the GM characteristics in common of C57Bl/6 mice was greater OB (>all other strains) and smaller cerebellar cortex (<BALB/cBy and C3H/He). The GM characteristics in common of BALB/cBy mice was neither greater nor smaller than those of any other strains or two strains. The GM characteristics in common of C3H/He mice was greater cerebellum (>all three other strains) and smaller OB and V1 (<C57Bl/6 and C3H/He). The GM characteristics in common of DBA/2 mice showed that none of the regions were greater than those of any other strains, but this strain had a smaller Au1 and CA1 (<all other strains).

## Discussion

The mouse is the most frequently employed experimental animal in studies of genetics, neuroscience, immunology, cancer, and medical and biological fields. Therefore, non-invasive and automatized phenotyping tools for entire mouse brain morphology are increasingly attractive. Recent advances in the sophistication of MRI hardware currently enable image assessment from mice and other small experimental animals at a resolution of several tens of microns^20, 21^. In addition, VBM is an attractive tool to use when making the transition from human to small animal imaging. In this study, we extended VBM analysis to the mouse by creating an *in vivo* tissue-segmented brain template of the four major inbred strains. We then performed VBM analysis of *in vivo* microscopic MRI data obtained from these strains to elucidate differences in brain morphology among them.

Brain templates have been published for humans, non-human primates, and rodents^6, 14, 22–24^. These brain templates permit the performance of advanced brain mapping techniques, including the above-described VBM, as well as functional-MRI and positron emission tomography.

In the case of automated morphometry of the mouse brain, although the registration methods based on the deformation field are widely used because of the precise registration of the lissencephalic brain^25–29^, no software has been designed specifically for the mouse brain. VBM with DARTEL in SPM8 enables the use of a diffeomorphic algorithm and notably improves the registration^30^. Here, we created TPMs for the mouse brain that are suited to SPM8. This in turn allows deformation-based morphometry to be conducted on mice using SPM8. Furthermore, evaluation of volume changes is directly possible from calculations of the Jacobian determinant obtained from VBM-DARTEL.

Segmented tissue templates (GM, WM, and CSF) of *ex vivo* mouse brain MRI data were previously published on the web by Sawiak *et al.* ^6^; we took advantage of these templates and used them as probability maps for the initial step of the segmentation procedure for the *in vivo* brain MRI data. However, an *in vivo* brain template is preferable for precise segmentation of the *in vivo* data because obvious shape deformations exist (the volume of the whole brain decreased by 10.6% and that of the ventricles decreased by 78.6%) in the *ex vivo* brain, as well as differences in image contrast over most of the *in vivo* versus *ex vivo* brain; this is due to intracranial brain pressure (*in vivo* brain images) and chemical fixation procedures (*ex vivo* brain images)^24, 31^.

Additionally, in the case of VBM, skull-stripping is required to extract the brain region from the whole head image, either by manual segmentation or by using automatic software (BET^17^ or the segmentation function in SPM8, with TPMs for the human brain). However, different brain morphology for mice and humans (e.g., there are smaller spaces between the brain parenchyma and the skull in mice), as well as the relatively elongated mouse brain, result in erroneous murine brain areas when using BET. For this reason, skull-stripping in rodent studies was previously conducted using a labor-intensive manual method. Some groups previously proposed semi-automatic or automatic skull-stripping of the rodent brain^32–35^. However, the approach used herein allows fully automated skull-stripping using only SPM software and our TPMs, which are freely available (http://www.nitrc.org/projects/tpm_mouse), without the need for dedicated programs or specialized technical knowledge. We also uploaded the templates for BALB/cBy, C3H/He, and DBA/2 mice; these templates could be useful in precise voxel-wise analyses, including automatic skull-stripping for each strain, as the four strains exhibited many differences in brain structure. Furthermore, these templates will be useful for site-target operations in the brain (e.g., implantation of cells, electrodes, injection of chemical substances) in addition to photomicrographic atlas, because these new templates have been constructed using *in vivo* high resolution MRI data in stereotaxic coordinates.

The International Mouse Phenotyping Consortium was created with the intention of ascribing a role to each gene in the mouse genome by 2021 by using 20,000 knock-out mouse lines, and the demand for normalized phenotype analysis is increasing. Considerations of model mouse phenotypes range from morphological analysis to behavioral assessment to evaluation of physiological function. A comprehensive investigation of region-specific differences would provide a guidepost for subsequent investigations and contribute to our systematic understanding of strain-specific brain characteristics. Previously, disparities in brain structure among various mouse strains were investigated by using MRI ROI analysis in C57Bl/6, DBA/2, and nine BXD recombinant inbred mice *ex vivo* ^10^. Badea *et al.* revealed that DBA/2 mice had a smaller brain volume than C57Bl/6 mice, and their observation that the volume of the hippocampus in DBA/2 mice is small is consistent with ours. Penet *et al.* investigated C57BL/6, FVB/N, and 129/SvJ mice *in vivo* (Penet *et al.*, 2006) and Chen *et al.* evaluated differences in brain structure in 129S1/SvImJ, C57Bl/6, and CD1 mice by applying VBA to *ex vivo* mouse brains^36^. To the best of our knowledge, however, this study is the first to characterize differences in brain morphology among mice strains by applying *in vivo* microscopic VBA including VBM.

The C57Bl/6 mouse is the most popular mouse strain and is widely used as a genetically modified animal model. We found that C57Bl/6 mice had GM characteristics consistent with a greater OB and a smaller cerebellar cortex. Mirich *et al.* performed a histological analysis of OB differences between C57Bl/6, BALB/cBy, and DBA/2 mice, and found a tendency for C57Bl/6 to have a greater OB volume and larger astrocytes with a more dense distribution^37^. It is known that the cerebellum of C57Bl/6 mice shows a relatively simple folial pattern compared with that in other strains^38^. Also, a strain comparison study of mouse cerebellum, including that of strains C57Bl/6 and BALB/cBy, revealed that the cerebellum volume of C57Bl/6 mice is below average, and smaller than that of BALB/cBy mice^39^.

The C3H/He mouse is an inbred mouse used to study cancer, infection, and sensorineural phenomena, among others^40^. It is widely known that the C3H/He strain carries a homozygous Pde6b^rd1^ mutation that causes retinal degenerative disease, and the mice become blind by the age of weaning^41^. Hence, our VBM observations of a smaller GM volume in the V1 of C3H/He mice may reflect a cortical volume reduction associated with the loss of vision in these animals.

The DBA/2 mouse is widely used in epilepsy, radiation, and hearing research^42–44^. It is also widely known that this strain carries three recessive alleles (Cdh23^ahl^ and two others) that cause a progressive disorder of the cochlea, and the mice show severe hearing loss by 3 months of age^45^. We found a smaller GM volume in the Au1 of the DBA/2 mouse, which may reflect the diminished auditory function in this strain. The DBA/2 mouse also demonstrates low spatial learning capacity at the age of >24 days relative to the C57Bl/6 mouse, in spite of normal function at 17 days^46^. Regarding physiological function, low long-term potentiation in the CA1 is essential to the spatial learning process^47^. In temporal lobe epilepsy patients, a smaller hippocampal volume is observed with VBM^48^, and these patients also marked notable memory deficits^49^. Our demonstration of a smaller GM volume in the CA1 of DBA/2 mice may, therefore, also be related to audiogenic epilepsy in the DBA/2 strain. Although comparing the local GM volume of the entire brain among four inbred mouse strains revealed various strain-specific brain structures (other than the aforementioned area), the structural discrepancies responsible for cytoarchitecture and behavior remain obscure. Further investigations based on histological, functional and behavior analyses are required.

## Conclusions

Here, we created a brain template for the mouse to support the use of automatized VBM in mouse models. Furthermore, we successfully characterized inbred mouse strains by elucidating inter-strain differences in brain morphology. Based on our observations, we propose that the use of automated mouse VBM, as established herein, will contribute to the investigation of mouse brain phenotypes.

## Electronic supplementary material

Table S1
