# Supplementary material for: In vivo microscopic voxel-based morphometry with a brain template to characterize strain-specific structures in the mouse brain
Source: Sci Rep. 2017 Mar 7;7:85. doi: 10.1038/s41598-017-00148-1 (PMC5427914; doi:10.1038/s41598-017-00148-1)
Supplement: Supplementary file 1 — Table S1 [file 41598_2017_148_MOESM1_ESM.pdf]

Supplementary Materials for

In vivo microscopic voxel-based morphometry with a brain template to characterize strain-specific structures in the mouse brain

Keigo Hikishima, Yuji Komaki, Fumiko Seki, Yasuyuki Ohnishi, Hirotaka J. Okano, and Hideyuki Okano*

* Correspondence to: Hideyuki Okano (hidokano@a2.keio.jp)

This PDF file includes:

Table S1

Table S1. Gray matter volume differences according to brain region among four inbred mice strains

Significant differences in the gray matter volume of specific brain regions are shown, as identified via voxel-based morphometry ($p < 0.05$, family-wise error-corrected, >119 voxels).

C57Bl/6 > BALB/cBy

Cluster size	Anatomical location	X	Y	Z	Z-scores
2961	obolfactory bulb, right	−0.9	2.7	−3.7	5.8
	obolfactory bulb, left	1.0	2.8	−3.9	4.7
1051	subiculum transition area, right	−3.8	−4.6	−3.2	5.2
235	anteromedial thalamic nucleus, right	−0.6	−1.1	−4.0	5.1
551	primaly auditory cortex, right	−4.2	−3.2	−2.3	5.0

BALB/cBy > C57Bl/6

Cluster size	Anatomical location	X	Y	Z	Z-scores
340	lobules 3 of the cerebellar vermis, left	0.1	−6.0	−2.2	4.8

C57Bl/6 > C3H/He

Cluster size	Anatomical location	X	Y	Z	Z-scores
6039	olfactory bulb, right	−0.2	2.9	−3.7	7.4
1519	primaly visual cortex, right	−2.4	−5.2	−1.3	6.1
1590	primaly visual cortex, left	2.0	−5.2	−1.1	5.8
148	secondary somatosensory cortex, right	−4.5	−0.7	−3.3	5.2

C3H/He > C57Bl/6

Cluster size	Anatomical location	X	Y	Z	Z-scores
1360	lobules 6 of the cerebellar vermis, right	−0.8	−8.0	−1.7	6.3
	lobules 6 of the cerebellar vermis, left	0.7	−8.2	−1.8	6.1
259.0	simple louble, right	−2.2	−6.0	1.4	6.2
2863	lobules 2 and 3 of the cerebellar vermis, left	0.3	−6.0	−2.3	5.7
760	simple lobule, right	−2.6	−6.1	−2.9	5.5
713	simple lobule, left	2.3	−6.8	−2.0	5.1
365	crus 2 of the ansiform lobule, right	−2.7	−7.9	−3.1	5.1

C57Bl/6 > DBA/2

Cluster size	Anatomical location	X	Y	Z	Z-scores
21004	secondary somatosensory cortex, right	−4.4	−0.8	−3.3	7.3
	primaly auditory cortex, right	−4.1	−2.9	−2.5	6.7
	primaly somatosensory cortex, forelimb region, right	−2.2	0.8	−2.0	6.6
	primaly somatosensory cortex, barrel field, right	−3.4	−1.1	−1.8	6.3
	insular cortex, right	−2.8	1.5	−3.7	5.8
2143	field CA1 of the hippocampus, left	1.5	−3.2	−1.6	6.9
	dentate gyrus, left	2.9	−4.0	−2.8	5.9
2503	field CA1 of the hippocampus, right	−1.8	−3.6	−2.0	6.9
	dentate gyrus, right	−2.6	−4.0	−2.6	5.9
	dorsal subculum, right	−0.6	−2.5	−1.9	5.6
18973	primaly auditory cortex, left	4.6	−2.6	−2.1	6.8
	primaly somatosensory cortex, barrel field, left	3.4	−1.4	−1.4	6.6
	secondary somatosensory area, left	4.6	−1.0	−3.3	6.0
856	lateral orbital cortex, left	1.8	2.4	−2.5	6.0
658	insular cortex , left	2.6	1.6	−3.9	5.9
738	olfactory bulb	−0.2	3.0	−3.7	5.3

DBA/2 > C57Bl/6

Cluster size	Anatomical location	X	Y	Z	Z-scores
not detected					

BALB/cBy > C3H/He

Cluster size	Anatomical location	X	Y	Z	Z-scores
not detected					

C3H/He > BALB/cBy

Cluster size	Anatomical location	X	Y	Z	Z-scores
1096	simple lobule, left	2.5	−5.7	−3.2	6.3
5986	field CA3 of the hippocampus, right	−2.9	−3.0	−4.6	6.1
506	accumbens nucleus, core, left	0.8	1.3	−3.8	5.4
1055	anteromedial thalamic nucleus, right	−0.4	−1.1	−3.9	5.3
	anteromedial thalamic nucleus, left	0.2	−0.6	−3.9	5.1
219	lobules 6 of the cerebellar vermis, right	−0.7	−8.0	−1.7	5.2
1757	field CA3 of the hippocampus, left	2.3	−3.6	−4.5	5.1
573	simple lobule, right	−2.2	−5.8	−3.1	4.8
185	accumbens nucleus, core, right	−0.7	1.5	−3.7	4.7

BALB/cBy > DBA/2

Cluster size	Anatomical location	X	Y	Z	Z-scores
1302	temporal association cortex, right	−4.8	−4.0	−3.1	6.3
1475	field CA1 of the hippocampus, left	1.5	−3.3	−1.7	5.9
2251	accumbens nucleus, core, right	−1.4	0.5	−4.3	5.6
	ventral palludum, right	−1.9	−0.1	−4.9	4.9
776	field CA1 of the hippocampus, right	−1.8	−3.7	−1.9	5.3
865	primaly somatosensory cortex, barrel field, left	3.4	−1.4	−1.4	5.3
1010	primaly auditory cortex, left	4.6	−2.2	−2.1	5.3
192	primaly auditory cortex, right	−3.9	−2.5	−2.7	5.3
980	insular cortex, right	−1.9	2.2	−2.6	5.2
377	secondly auditory cortex, ventral area, right	−4.7	−2.0	−2.6	5.2
404	insular cortex, Left	1.8	2.3	−2.7	5.2

DBA/2 > BALB/cBy

Cluster size	Anatomical location	X	Y	Z	Z-scores
not detected					

C3H/He > DBA/2

Cluster size	Anatomical location	X	Y	Z	Z-scores
28989	field CA1 of the hippocampus, left	1.4	−3.2	−1.6	7.7
	dorsal subculum, left	0.6	−2.6	−1.6	7.7
	field CA1 of the hippocampus, right	−1.5	−3.4	−1.5	7.5
22101	secondary somatosensory cortex, left	3.8	0.8	−3.2	7.4
	primaly somatosensory cortex, left	3.1	1.6	−2.8	7.1
	insular cortex, left	2.7	2.1	−1.9	6.7
6867	simple louble, right	−2.5	−5.8	−2.4	7.4
	crus 1 of the ansiform lobule, right	−2.4	−6.0	−1.5	7.0
	lobules 6 of the cerebellar vermis, right	−0.8	−7.8	−1.7	6.6
28716	primaly somatosensory cortex, right	−3.4	1.2	−1.9	7.2
	frontal cortex, area3, right	−2.5	2.1	−1.9	7.1
	secondary somatosensory cortex, right	−3.8	0.8	−2.7	6.9
670	lobules 2 and 3 of the cerebellar vermis, left	0.3	−5.9	−2.8	5.5
250	primaly auditory cortex, left	4.4	−3.7	−2.8	5.1

DBA/2 > C3H/He

Cluster size	Anatomical location	X	Y	Z	Z-scores
717	obolfactory bulb, left	0.2	2.6	−3.8	5.2
	obolfactory bulb, right	−0.2	2.8	−3.0	4.6
